# Characterization of Carotenoid Biosynthesis in Newly Isolated *Deinococcus* sp. AJ005 and Investigation of the Effects of Environmental Conditions on Cell Growth and Carotenoid Biosynthesis

**DOI:** 10.3390/md17120705

**Published:** 2019-12-14

**Authors:** Jun Young Choi, Kunjoong Lee, Pyung Cheon Lee

**Affiliations:** Department of Molecular Science and Technology and Department of Applied Chemistry and Biological Engineering, Ajou University, Woncheon-dong, Yeongtong-gu, Suwon 16499, Korea; custum@ajou.ac.kr (J.Y.C.);

**Keywords:** *Deinococcus*, deinoxanthin, carotenoid

## Abstract

Our purpose was to characterize the structures of deinoxanthin from *Deinococcus* sp. AJ005. The latter is a novel reddish strain and was found to synthesize two main acyclic carotenoids: deinoxanthin and its derivative. The derivative (2-keto-deinoxanthin) contains a 2-keto functional group instead of a 2-hydroxyl group on a β-ionone ring. A deinoxanthin biosynthesis pathway of *Deinococcus* sp. AJ005 involving eight putative enzymes was proposed according to genome annotation analysis and chemical identification of deinoxanthin. Optimal culture pH and temperature for *Deinococcus* sp. AJ005 growth were pH 7.4 and 20 °C. Sucrose as a carbon source significantly enhanced the cell growth in comparison with glucose, glycerol, maltose, lactose, and galactose. When batch fermentation was performed in a bioreactor containing 40g/L sucrose, total carotenoid production was 650% higher than that in a medium without sucrose supplementation. The culture conditions found in this study should provide the basis for the development of fermentation strategies for the production of deinoxanthin and of its derivative by means of *Deinococcus* sp. AJ005.

## 1. Introduction

*Deinococcus* strains are Gram-positive bacteria having a variety of metabolic pathways. Their habitats range from common environments, such as air, soils, and seas [1,2,3], to extremes such as high altitudes: stratosphere and alpine conditions [4,5,6]. Novel *Deinococcus* strains have been continuously isolated from Antarctic and marine fishes in extreme environments [7,8]. Notably, *Deinococcus* strains are known for their survival under strong γ-rays or UV radiation and desiccation conditions [9]. Several studies have shown that, to withstand these extreme environments, *Deinococcus* strains have unique metabolic capabilities including redundant DNA repair systems and biosynthesis of antioxidants such as the carotenoid deinoxanthin [10,11].

The latter is a major monocyclic carotenoid present in many *Deinococcus* strains, and its chemical structure was first characterized using deinoxanthin from *D. radiodurans* featuring high resistance to radiation [12,13]. Deinoxanthin has been reported to be more effective in scavenging very reactive singlet oxygen species (which fatally damage cellular metabolic pathways) than β-carotene, lutein, and lycopene [14,15]. Furthermore, deinoxanthin has anticancer activity [16] and can serve as a biomarker of a living organism in space research [17]. Given that carotenoids, including deinoxanthin, are being used as antioxidants, cosmetic ingredients, and food or feed additives [18,19], carotenoid-producing *Deinococcus* strains have aroused interest as microbial producers of bioactive carotenoids.

**Figure 1:**
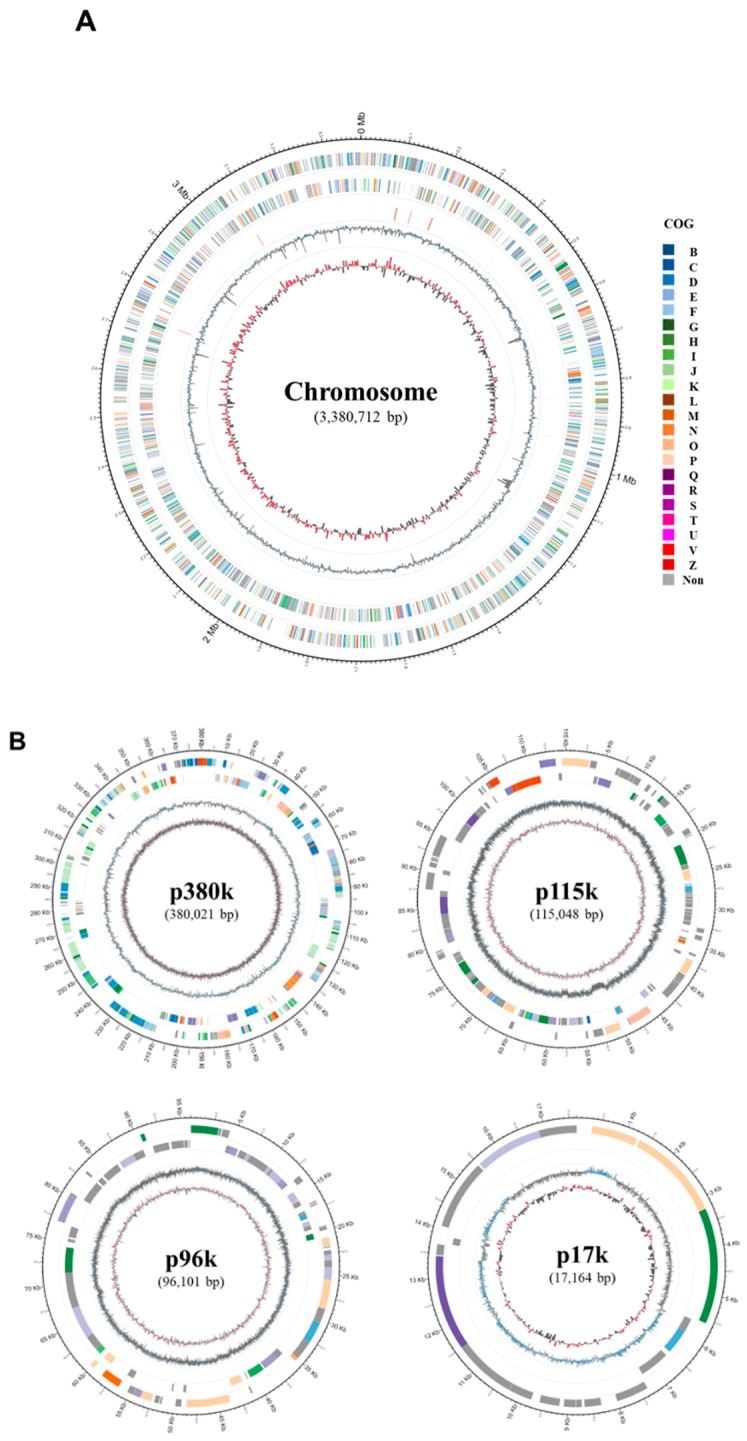
Circular representation of the chromosome and four plasmids of *Deinococcus* sp. AJ005. (**A**) From the outer to inner circle: predicted protein-coding sequences (colored according to Clusters of Orthologous Groups (COGs) categories) on the plus strand, predicted protein-coding sequences (colored by COGs categories) on the minus strand, RNA genes (transfer RNAs (tRNAs): blue, ribosomal RNAs (rRNAs): red), GC content (blue/black), and a GC skew (red/black). (**B**). From the outer to inner circle: predicted protein-coding sequences (colored by COGs categories) on the plus strand, predicted protein-coding sequences (colored by COGs categories) on the minus strand, GC content (blue/black), and a GC skew (red/black).

Recently, a novel reddish *Deinococcus* strain, AJ005, was isolated from seawater near King George Island, and the AJ005 genome was completely sequenced and made publicly available [20]. *Deinococcus* sp. AJ005 synthesizes red carotenoids, and its complete genome consists of a single circular chromosome (3.3 Mbp) and four circular plasmids (p380k, p115k, p96k, and p17k; Figure 1).

In this study, our purpose was to characterize the structures of deinoxanthin from *Deinococcus* sp. AJ005. We proposed that a deinoxanthin biosynthetic pathway exists in *Deinococcus* sp. AJ005 on the basis of genome annotation analysis and chemical identification of isolated carotenoids. In addition, we investigated the effects of culture conditions on the deinoxanthin biosynthesis in this strain. 

## 2. Results and Discussion

### 2.1. Characterization of Carotenoids of Deinococcus Sp. AJ005

**Figure 2:**
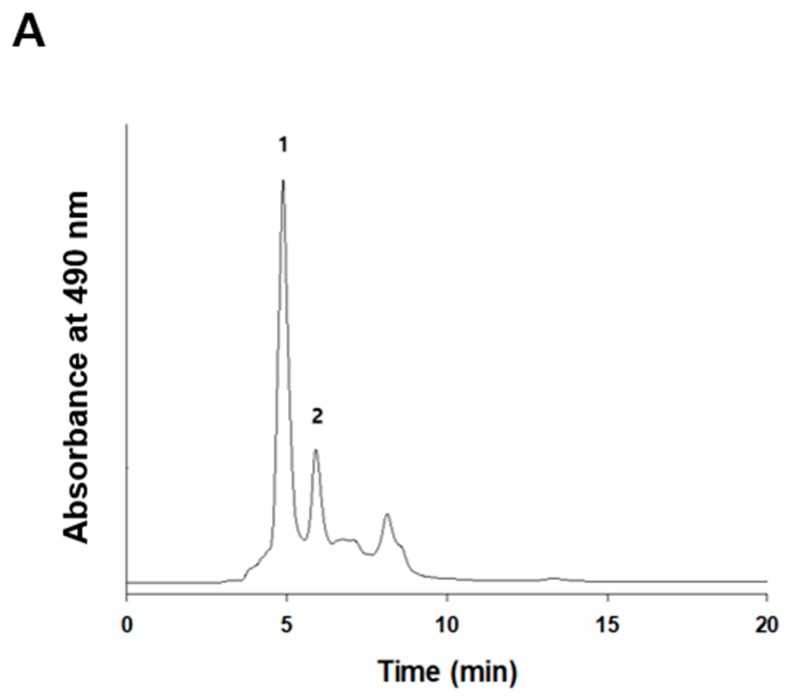

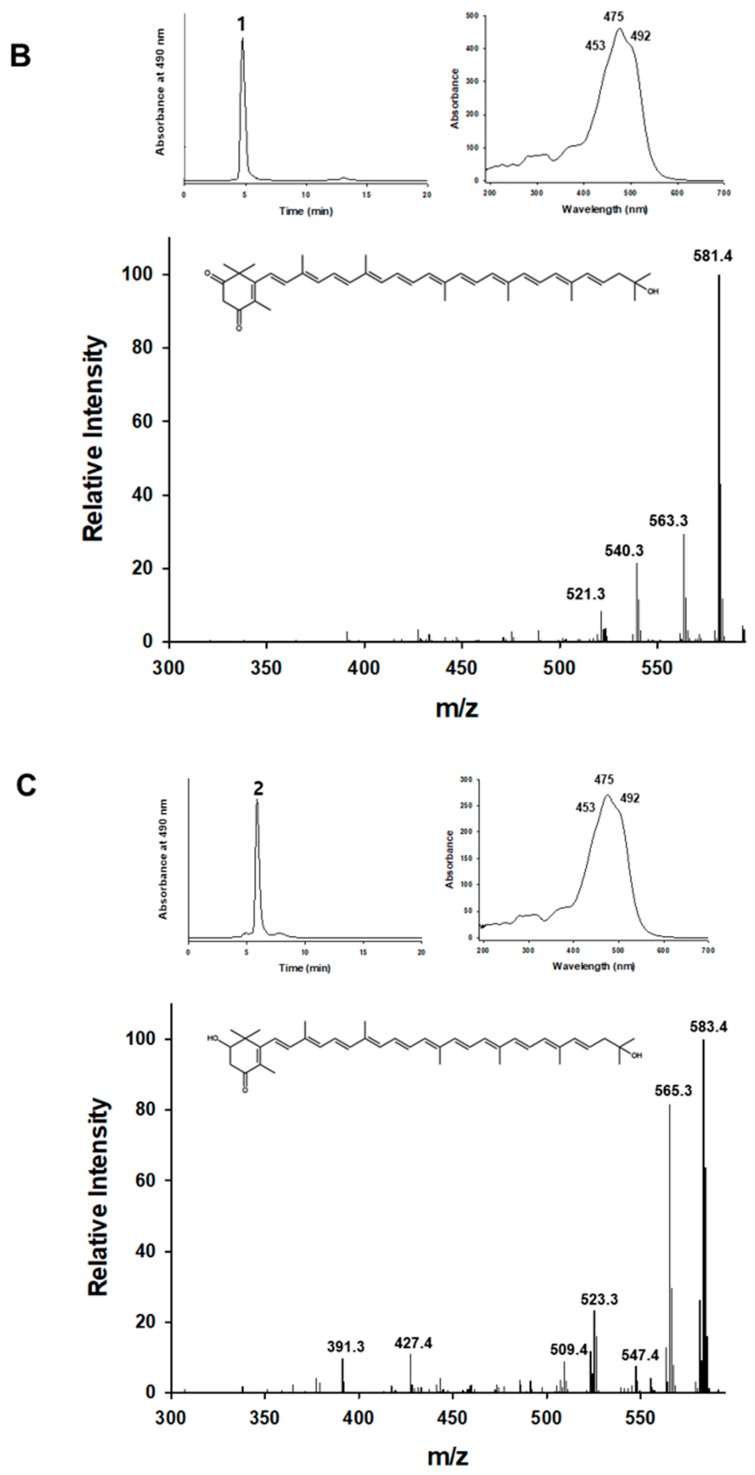
The carotenoid profile of *Deinococcus* sp. AJ005 and MS analysis of the carotenoids. (**A**) The high-performance liquid chromatography (HPLC) profile of a crude carotenoid extract; (**B**) HPLC/mass spectrometry (MS) chromatograms and the UV/Vis spectrum of the isolated deinoxanthin derivative; (**C**) HPLC/MS chromatograms and a UV/Vis spectrum of isolated deinoxanthin.

To identify carotenoids of *Deinococcus* sp. AJ005, total carotenoids were extracted and analyzed by C18 reverse-phase high-performance liquid chromatography (HPLC). The HPLC analysis of the carotenoid profile yielded two main polar peaks (Figure 2A). After purification by silica chromatography, the two polar carotenoids were analyzed by liquid chromatography with mass spectrometry (MS) in positive APCI (atmospheric-pressure chemical ionization) mode. According to our analysis of mass spectra and UV/Vis spectra, a carotenoid corresponding to peak 1 had a molecular ion (M+H)^+^ with m/z 581.4 and λ_max_ = 453 (shoulder), 475, and 492 (shoulder) nm (Figure 2B), whereas the carotenoid corresponding to peak 2 had a molecular ion (M+H)^+^ of m/z 583.4 and λ_max_ = 453 (shoulder), 475, and 492 (shoulder) nm (Figure 2C). The mass fragmentation pattern of peak 2 was similar to the reported pattern of deinoxanthin [21,22]: 565.3 ((M+H)^+^ − 18; loss of H_2_O), 547.4 ((M+H)^+^ − 18 − 18; loss of 2 molecules of H_2_O), and 523.3 ((M+H)^+^ − 60; loss of the acyclic end containing a hydroxyl group). On the basis of the molecular ion, UV/Vis spectra, and the mass fragmentation pattern, the carotenoid corresponding to peak 2 is proposed to be deinoxanthin. The carotenoid corresponding to peak 1 had the same UV/Vis spectrum as that of deinoxanthin and a mass fragmentation pattern similar to that of deinoxanthin with a difference of −2 m/z: 561.3 ((M+H)^+^ − 18) in peak 1 versus 565.3 ((M+H)^+^ − 18) in peak 2 and 521.3 ((M+H)^+^ − 60) in peak 1 versus 523.3 ((M+H)^+^ − 60) in peak 2. Therefore, the carotenoid corresponding to peak 1 is proposed to be a deinoxanthin derivative (2-keto-deinoxanthin) containing a 2-keto functional group instead of a 2-hydroxyl functional group in the ring structure. The 2-keto group in 2-keto-deinoxanthin did not significantly influence the shape of the UV/Vis spectrum, resulting in the same UV/Vis spectrum of deinoxanthin. 

### 2.2. The Proposed Carotenoid Biosynthetic Pathway and Genes Encoding Putative Carotenogenic Enzymes in Deinococcus Sp. AJ005

**Figure 3:**
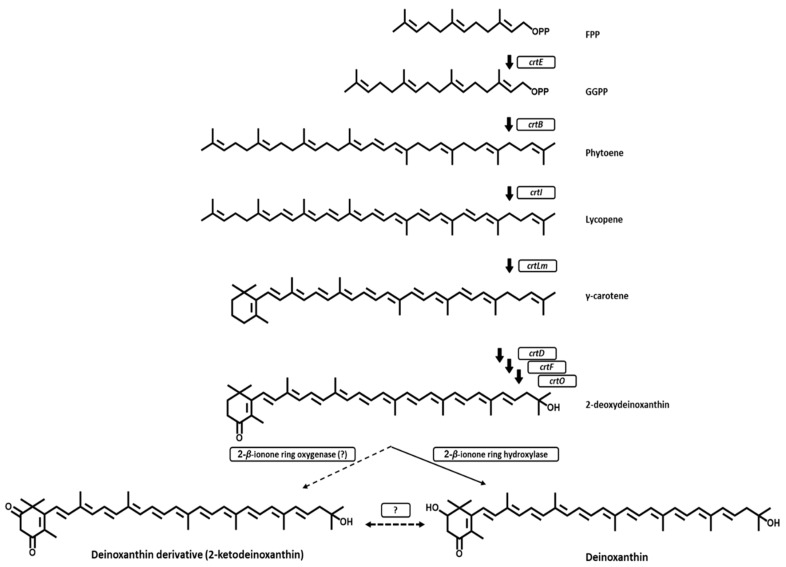
Proposed biosynthetic pathways for deinoxanthin and for its derivative in *Deinococcus* sp. AJ005^T^. The following enzymes are involved in these biosynthetic pathways: CrtE, GGPP synthase; CrtB, phytoene synthase; CrtI, phytoene desaturase; CrtLm, lycopene cyclase; CrtO, β-carotene 4-ketolase; CruF, C-1’,2’ hydratase; CrtD, C-3’,4’ desaturase; 2-β-ionone ring hydroxylase; and 2-β-ionone ring oxygenase (unidentified). The ? mark represents the possibility of oxido-reduction catalyzed by an unknown enzyme.

Genome annotation analysis predicted seven genes encoding the putative deinoxanthin pathway enzymes on the chromosome of *Deinococcus* sp. AJ005: GGPP synthase (*crtE*), phytoene synthase (*crtB*), phytoene desaturase (*crtI*), lycopene cyclase (*crtLm*), β-carotene 4-ketolase (*crtO*), C-1’,2’ hydratase (*cruF*), and C-3’,4’ desaturase (*crtD*). Unfortunately, a gene encoding 2-β-ionone ring hydroxylase for the deinoxanthin biosynthesis and a gene encoding 2-β-ionone ring oxygenase for the biosynthesis of the deinoxanthin derivative (2-keto-deinoxanthin) were not found. Nonetheless, there was a gene encoding a putative cytochrome P450 (which might be a 2-β-ionone ring hydroxylase) on the chromosome of *Deinococcus* sp. AJ005. A recent study [22] showed that the cytochrome P450 CYP287A1 of *D. radiodurans* R1 is a novel 2-β-ionone ring hydroxylase in the deinoxanthin pathway. The BLASTp analysis revealed that there is a 70% amino acid identity between the putative cytochrome P450 of *Deinococcus* sp. AJ005 and the cytochrome P450 CYP287A1 of *D. radiodurans* R1. In addition to the above eight genes, one gene coding for a putative lycopene elongase (*crtEb*), two genes encoding a putative phytoene dehydrogenase, two genes encoding a putative cytochrome P450 hydroxylase, and one gene encoding a possible cytochrome P450 were predicted on the chromosome of *Deinococcus* sp. AJ005. According to the chemical identification of deinoxanthin and of the deinoxanthin derivative from the crude carotenoid extract of *Deinococcus* sp. AJ005 and according to the putative carotenogenic enzymes, a carotenoid pathway of *Deinococcus* sp. AJ005 was proposed (Figure 3). Functional analysis of the putative enzymes (in particular three cytochrome P450s) needs to be performed to elucidate the proposed carotenoid pathway.

### 2.3. Effects of Culture Media, Culture pH, and Temperature on Cell Growth

**Figure 4:**
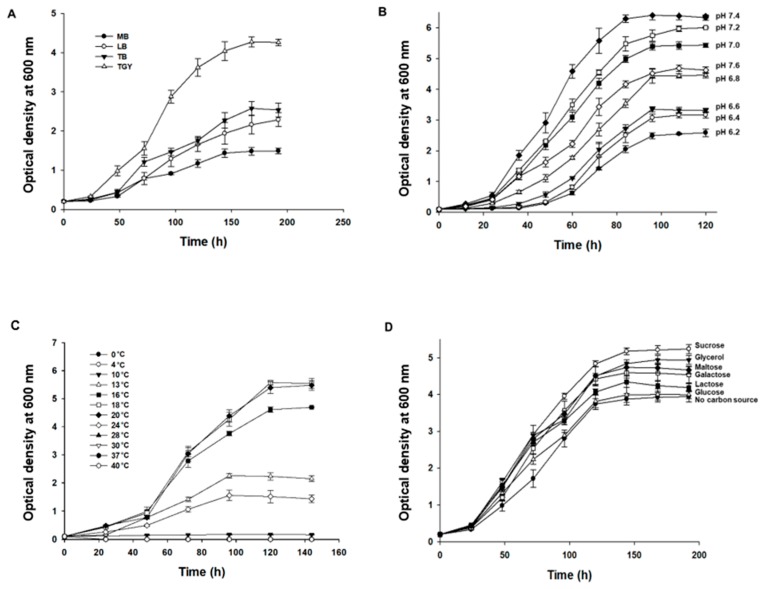
Cell growth of *Deinococcus* sp. AJ005^T^ in flask cultures under different culture conditions. (**A**) Four culture media (pH 7.5 and 20 °C); (**B**) eight culture pH values (20 °C); (**C**) 12 culture temperatures (pH 7.4); and (**D**) six carbon sources (10 g/L, 20 °C, and pH 7.4).

To investigate the effects of culture media, culture pH, temperature, and a carbon source on the growth of *Deinococcus* sp. AJ005 cells, 100 mL flask cultures were carried out. Among several media (Luria–Bertani (LB), Terrific Broth (TB), Marine Broth 2216 (MB), and Tryptone-Glucose-Yeast extract (TGY) broth) containing 1 g/L glucose and with pH adjusted to 7.5, the growth of *Deinococcus* sp. AJ005 cells reached the highest OD_600_ of 4.3 ± 0.1 in the TGY medium at 20 °C, followed by the TB, LB, and MB media (Figure 4A). Next, the effects of culture pH and temperature on the growth of *Deinococcus* sp. AJ005 cells were studied in the TGY medium containing 1 g/L glucose. An initial culture pH of 7.4 at 20 °C was found to be optimal for cell growth (OD_600_ of 6.4 ± 0.1), and pH values above 7.6 or below 6.8 significantly reduced the cell growth (Figure 4B). At culture temperatures of 18 °C and 20 °C, the cell growth reached the highest OD_600_ of 5.5 ± 0.3 in the TGY medium containing 1 g/L glucose and with pH adjusted to 7.4. No growth was observed below 4 °C or above 28 °C (Figure 4C). Carbon sources significantly affected the growth of *Deinococcus* sp. AJ005 cells. When *Deinococcus* sp. AJ005 was grown in the TGY medium containing 10 g/L one of six carbon sources (i.e., glucose, glycerol, maltose, lactose, galactose, or sucrose); the greatest cell growth (OD_600_ of 5.3 ± 0.2) was observed in the TGY medium containing sucrose, followed by glycerol, maltose, galactose, lactose, and glucose (Figure 4D).

### 2.4. Batch Fermentation Involving Deinococcus sp. AJ005 for Carotenoid Production

**Figure 5:**
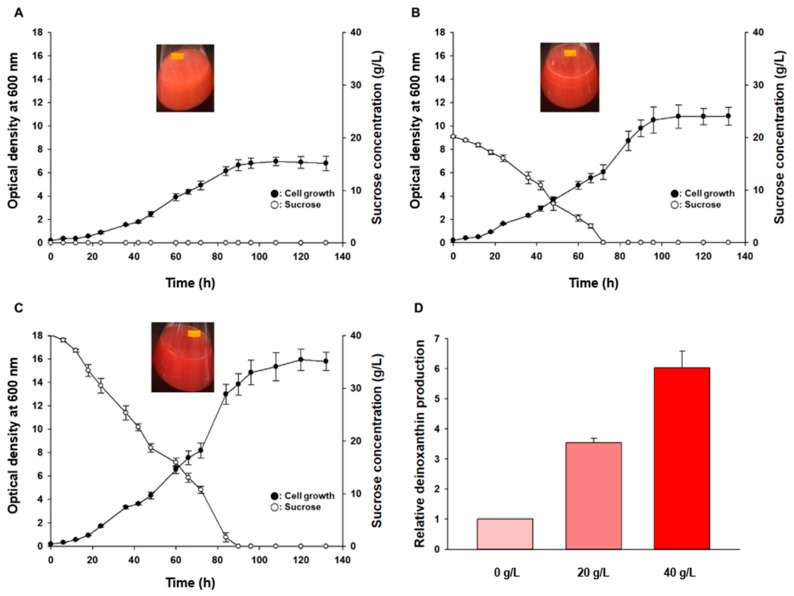
Bioreactor batch fermentation of the TGY(medium (containing different concentrations of sucrose) by *Deinococcus* sp. AJ005. (**A**) 0 g/L sucrose (**B**); 20 g/L sucrose; (**C**) 40 g/L sucrose; (**D**) relative carotenoid production in the medium containing 0, 20, or 40 g/L sucrose.

To achieve high production of deinoxanthin and of the deinoxanthin derivative, bioreactor fermentation with *Deinococcus* sp. AJ005 was performed at 20 °C and pH 7.4 in the TGY medium containing 0, 20, or 40 g/L sucrose. In the TGY medium containing 0 g/L sucrose, the cell growth reached an OD_600_ of 7.3 ± 0.3, and the maximum specific growth rate was 0.085 h^−1^ (Figure 5A). In the TGY medium containing 20 g/L sucrose, the cell growth reached an OD_600_ of 11.2 ± 0.7, and the maximum specific growth rate was 0.12 h^−1^ (Figure 5B). We noticed that 20 g/L sucrose was completely consumed in 72 h. In the TGY medium containing 40 g/L sucrose, the cell growth reached an OD_600_ of 16.1 ± 0.6, and the maximum specific growth rate was 0.18 h^−1^ (Figure 5C), whereas 40 g/L sucrose was completely consumed in 90 h. Total carotenoid production was proportional to the cell growth (Figure 5D). Total carotenoid production in the TGY medium containing 40 g/L sucrose was 650% higher than that in the TGY medium containing 0 g/L sucrose and 80% higher than that in the TGY medium containing 20 g/L sucrose.

## 3. Materials and Methods 

### 3.1. Flask Fermentation Involving Deinococcus Sp. AJ005

*Deinococcus* sp. AJ005, isolated from seawater near King George Island, was grown in 100 mL of a culture medium in a 500 mL baffled flask with rotary shaking at 250 rpm at various pH levels and temperatures as described below. Luria-bertani, TB, MB, and TGY media containing 1 g/L glucose were used for growing *Deinococcus* sp. AJ005 at 20 °C and pH 7.5. To investigate the influence of culture pH on the cell growth, *Deinococcus* sp. AJ005 was grown in 100 mL of the TGY medium containing 1 g/L glucose and with pH adjusted to 6.2–7.6 in increments of 0.2 by means of the phosphate buffer system. To investigate the impact of culture temperature on the cell growth, *Deinococcus* sp. AJ005 was grown at 0, 2, 4, 10, 13, 16, 18, 20, 24, 28, 30, 37, or 40 °C in 100 mL of the TGY medium containing 1 g/L glucose and with pH adjusted to 7.4. Glucose, glycerol, maltose, lactose, galactose, and sucrose (Sigma–Aldrich) served as a carbon source (10 g/L) in the TGY medium with pH adjusted to 7.4.

### 3.2. Bioreactor Fermentation by Deinococcus Sp. AJ005

Bioreactor batch fermentation was carried out with 1.5 L of the TGY medium containing sucrose (0, 20, or 40 g/L) as a carbon source in 5 L jar BioFlo 320 (Eppendorf, Hamburg, Germany). Pre-cultures (100 mL) were grown in the TGY medium at 20 °C for 1 day and inoculated into a bioreactor. The temperature was maintained at 20 °C, and pH was automatically maintained at 7.4 by adding 2.0 N HCl or 2.0 N NaOH. The dissolved oxygen level was maintained at 30% by supplying air and by adjusting agitation between 200 and 500 rpm.

### 3.3. Extraction and Analysis of Carotenoids

*Deinococcus* sp. AJ005 was grown in 1 L of the TGY medium. Carotenoids were repeatedly extracted from cell pellets of *Deinococcus* sp. AJ005 by means of 10 mL of methanol. The colored supernatants were concentrated to 10 mL in an EZ-2 Plus centrifugal evaporator (Genevac Inc., Ipswich, United Kingdom), and an equal volume of ethyl acetate and of a 5N NaCl solution was added. The upper colored phase was collected, washed with distilled water, passed through an anhydrous magnesium sulfate column, and completely dried with nitrogen gas. Carotenoids were purified from the crude carotenoid extract by silica chromatography (10 × 50 cm) based on silica gel 60 (Merck, Darmstadt, Germany). Elution was performed with hexane:acetone (6.5:3.5 *v*/*v*). Each purified carotenoid was completely dried and dissolved in methanol. Next, 10 μL of a carotenoid was injected into an Agilent 1200 HPLC system equipped with a ZORBAX SB-C18 column (4.6 × 250 mm, 5-micron, Agilent Technologies, Santa Clara, California, USA) and with a photodiode array detector (Agilent Technologies, USA). Each carotenoid was subjected to diode array detection at 480 nm wavelength. The HPLC conditions were as follows: 23 °C column temperature, a 0.8 mL/min flow rate, and an isocratic mobile phase of acetonitrile, methanol, and isopropyl alcohol (40:50:10 *v*/*v*/*v*). The mass fragmentation spectra of carotenoids were monitored in the positive ion mode of an Agilent LC/MS 6150 Quadrupole system equipped with an atmospheric-pressure chemical ionization interface (Agilent Technologies). The MS conditions were as follows: 3 kV capillary voltage, 4.0 μA corona current, 12 L/min drying gas flow, 35 psi_g_ nebulizer pressure, and 350 °C drying gas temperature and vaporizer temperature. 

### 3.4. Monitoring Cell Growth and Quantification of Carbohydrates and Carotenoids

Cell growth was monitored by measuring the absorbance at 600 nm (OD_600_) with a SPECTRAmax Plus384 spectrophotometer (Molecular Devices, San Jose, California, USA). The concentrations of glucose, glycerol, maltose, lactose, galactose, and sucrose were determined using an Agilent 1100 HPLC system equipped with a refractive index detector (Agilent, Santa Clara, California, USA) and an Aminex HPX-87H column (Bio-Rad, Hercules, California, USA) at a flow rate of 0.7 mL/min and column temperature of 50 °C, with 4 mM H_2_SO_4_ as the mobile phase. The total-carotenoid amount was measured via the absorbance of the methanol extract at a wavelength of 475 nm on the SPECTRAmax Plus384 spectrophotometer.

## 4. Conclusions

*Deinococcus* sp. AJ005 synthesizes two main types of acyclic carotenoids: deinoxanthin and a deinoxanthin derivative (2-keto-deinoxanthin). Genome sequence analysis of deinoxanthin-producing *Deinococcus* sp. AJ005 uncovered eight genes encoding putative deinoxanthin biosynthesis enzymes. The culture conditions found in this study should provide the basis for the development of fermentation strategies for the production of deinoxanthin and of its derivative by means of *Deinococcus* sp. AJ005.
